# Transmission Dynamics of *Corynebacterium* spp. Within Two Danish Dairy Cattle Herds

**DOI:** 10.3389/fvets.2021.735345

**Published:** 2021-08-23

**Authors:** Carsten Kirkeby, Tariq Halasa, Michael Farre, Galal Nazih Chehabi, Kaare Græsbøll

**Affiliations:** ^1^Department of Veterinary and Animal Sciences, Faculty of Health and Medical Sciences, University of Copenhagen, Copenhagen, Denmark; ^2^SEGES Livestock Innovation, Aarhus, Denmark; ^3^Technical University of Denmark (DTU) Compute, Technical University of Denmark, Kongens Lyngby, Denmark

**Keywords:** *Corynebacterium*, dairy cows, mastitis, intramammary infection, transmission dynamics

## Abstract

Intramammary infections (IMI) can cause mastitis, a prevalent and costly infectious disease in dairy cattle worldwide. The IMI is caused by a range of bacteria, including *Corynebacterium* spp. Knowledge of the transmission dynamics of pathogens is generally sparse but essential to support decision-making; such as input to bioeconomic models. In this observational study, we explored the transmission dynamics of *Corynebacterium* spp. in two different Danish dairy cattle herds by testing monthly quarter-level milk samples of all lactating cows for 1 year. We estimated the prevalence for herd 1 and 2 to 24 and 11.7%, respectively, and the mean quarter-level incidence to be 8 and 6.5% per month, respectively. We compared a model for indirect transmission via the environment with a model with the direct contagious transmission and found that the latter model best explained the data. We estimated the daily mean quarter-level transmission rate to be 0.016 and 0.018 cases/quarter-day for herd 1 and 2, respectively. The mean recovery rate was 0.012 and 0.016 for herd 1 and 2, respectively. Consequently, the basic reproduction number for herd 1 and 2 was 1.27 and 1.10, respectively. This study highlights that *Corynebacterium* spp. can be prevalent within a herd and transmit directly between cows. Thus, future studies should investigate cost-effective control measures against *Corynebacterium* spp.

## Introduction

Intramammary infection (IMI) causes mastitis worldwide in dairy herds, decreasing animal welfare, and causing economic losses to farmers through reduced milk production (1). IMI is mainly caused by bacteria traditionally classified as either contagious or environmental, based on whether they are directly transmitted between the cows or come from a reservoir in the environment (2). *Corynebacterium* spp. are traditionally classified as an environmental pathogen, even though the predominant transmission mode is unknown (3, 4). *Corynebacterium* spp. are also often classified as minor mastitis pathogens associated with reduced milk production (5). In recent years, attention has increased toward intramammary infections (IMI) caused by *Corynebacterium* spp. in dairy herds (4, 6–9). Examples of species that have been observed causing mastitis in dairy cows are *Corynebacterium pyogenes* (10) and *Corynebacterium pseudotuberculosis* (11). *Corynebacterium* spp. can cause clinical and subclinical mastitis and result in substantial production losses, mainly when infections occur before peak production (12).

The spread of IMI-causing pathogens among dairy cows can be prevented by regular management strategies, including post-milking teat dipping, antimicrobial treatment, and culling of infected cows (13). Simulation models help estimate the optimal strategy in each herd (14). To simulate cost-effective control strategies for *Corynebacterium* spp., an investigation of the transmission dynamics of *Corynebacterium* spp. in dairy herds through field studies is needed. Understanding and quantifying the pathogen transmission can help developing reliable decision support tools. To our knowledge, only one study has described the transmission dynamics of IMI caused by *Corynebacterium* spp. in U.S. dairy cows (8). To investigate the variance in the transmission dynamics of *Corynebacterium* and be able to include it in decision support tools, it is essential to obtain estimates of the transmission dynamics from more herds and other geographical regions.

This study aimed to investigate the transmission dynamics of *Corynebacterium* spp. in two Danish dairy herds using data from a longitudinal 12-month survey. Firstly, we wanted to examine whether transmission of *Corynebacterium* spp. is best described as a fixed rate of infection from an environmental reservoir or if contagious transmission occurs directly between cows e.g., during the milking process. Furthermore, we wanted to estimate the transmission and recovery rates and the basic reproduction number. We used three different methods to examine the robustness of the results.

## Materials and Methods

### Animals and Farms

We used data of monthly quarter level milk samples from all lactating cows in two Danish dairy herds throughout 1 year between January 2017 and January 2018. The herds had an average number of milking cows of, respectively, 180 and 360 cows and both herds had side-by-side milking parlors. Herd 1 was milked two times per day, and Herd 2 was milked three times per day. The bedding material in both herds was straw. Herd 1 had a mean bulk milk SCC of 294,000 (range = 261,000–324,000 cells/mL) and Herd 2 had a mean bulk milk SCC of 280,000 (range = 236,000–299,000 cells/mL) (15). We selected the length of the sampling interval to mimic the routine performance samples that take place approximately 11 times annually (Dairy Herd Improvement sampling). In total, we sampled 214, and 443 lactating cows from Herd 1 and 2, respectively (Table 1). A detailed description of the herds and the sampling procedure can be found in Kirkeby et al. (15).

**Table 1:** Key characteristics of the two study herds.

	**Herd 1**	**Herd 2**
Herd size (cows)	180	360
Average daily milk production per cow (kg)	30.4	39.7
Bulk milk SCC (cells/mL)	294,000	280,000
Breed	Holstein	Holstein
Bedding material	Straw	Sand
Milking parlor	12 × 2 side- by-side	16 × 2 side-by-side

In Herd 1, the cows were housed in free stalls with mattresses, and straw was used as bedding material. Lactating cows were milked two times a day in a parallel parlor of 2 × 12 cows. The procedure for milking was according to the NMC recommendations using gloves and applying pre and post-dip. In Herd 2, the lactating cows were milked three times a day also in a parallel parlor, here in 2 × 16 cows. At milking, the personnel used gloves, and teats were wiped off using clean cotton towels, but no post-milking teat dipping was implemented.

### Collection of Quarter Milk Samples

The collection of quarter milk samples was performed in line with NMC standards (http://www.nmconline.org/sampling.htm). As described in Kirkeby et al. (15), the teats in Herd 1 were sprayed with a foaming teat wipe-off product (Viri Foam,Novadan ApS, Kolding, Denmark), whereas the teats in Herd 2 were predipped using milk wash from Trinol (Hobro, Denmark). The teats were then cleaned with cotton towels soaked in water and a minimum of 4 squirts of milk were discarded from each quarter. After this teats were sanitized using single-service wet wipes (MS Lavettes, MS Schipper, Bladel, the Netherlands) soaked in 90% ethanol, with one towel per teat. The teats were further cleaned before milk sampling using 90% ethanol spray followed by air-drying for a minimum of 30 s. For sampling, pre-labeled sterile sample vials were applied. After filling of the vials with 15 ml milk, they were recapped immediately. All samples were stored in thermally insulated boxes, with cooling elements shipped to the laboratory. The samples were then preserved using 0.5% boric acid, and bacteriological analysis took place within 48 h of collection. Bacteriological analysis was conducted as explained in Kirkeby et al. (16). Briefly, identification was performed using bacteriological culture (BC) where a sterile glass loop was used to streak 10 μLof each sample to a quarter of a blood agar plate with 0.1% esculine, corresponding to one plate per cow. Then followed aerobic incubation at 37°C for up to 48 h. A positive sample was identified if there was minimum 1 colony. Pathogens were identified by morphology under a microscope. Final confirmation was carried out using MALDI-TOF MS using Biotyper Version V3.3.1.0 (DB 5989, Bruker Daltonik, 2012). Contaminated samples were determined if there were more than three different pathogens present. The farmers did not receive information on the results and did therefore not change their management procedures in response to any findings.

### Statistical Analysis

The statistical analysis was conducted in R 3.6.3 (17), and figures were generated using the package ggplot2 (18). We obtained data on the dates for dry-off per cow during the study period from the Danish cattle database. We estimated quarter-level prevalence and incidence per sampling interval for both herds. We corrected missing data points to increase data size by applying the following rules (15). A missing value between two positive samples was corrected to positive; a missing value between two negative samples was corrected to negative; and missing values between any negative and positive samples were corrected to negative. Additionally, if a missing value followed two positive samples, we corrected it to be positive (15). We considered infections to be recovered once a negative sample occurred after a positive sample and no positive sample followed that. The recovered animals were either spontaneously recovered or treated with antimicrobials. Data from cows after treatment was excluded from this study.

### Estimating Transmission and Recovery Rates

Poisson regression is a commonly applied method for estimating transmission rates in both epidemic and endemic scenarios (8, 19). We here wanted to assess and compare the transmission rates of *Corynebacterium* spp. using three different methods, namely Poisson regression and two other methods (20). One advantage of these methods is flexibility because longitudinal field studies can include many introductions and exits of animals between samplings. These methods are less sensitive to these effects than the Poisson regression. Furthermore, Poisson regression and method 1 are sensitive to the time between sampling intervals because they do not consider reinfections between sampling. Method 2 takes this into account, correcting for the possibility of multiple infections between samplings (20).

To estimate the transmission rate of *Corynebacterium* spp. with Poisson regression, we used a model with a log-link, assuming that each newly infected quarter is infected halfway between two sampling points, as described in Zadoks et al. (19):

(1) $$log\hat{(}I_{N})=\log(\beta )+\log\left(\frac{S_{int}\cdot I_{int}}{N_{int}}\right)$$

where log(IN)^ Indicates the expected log number of newly infected quarters at each sampling interval, *β* is the estimated transmission rate of *Corynebacterium* spp. *S*_*int*_ and *I*_*int*_ are the number susceptible and infectious quarter days at risk, respectively. N_int_ is the total number of susceptible and infectious quarter days at risk. We used the term log(*S*_*int*_ · *I*_*int*_/*N*_*int*_) as an offset in the regression analysis, meaning that this model reflects contagious transmission directly between cows. Like in Dalen et al. (8), we also constructed a similar model with log(*S*_*int*_) as the offset, they mimic a fixed rate of infection, independent of the proportion of infected animals present at the sampling points. This model reflects environmental transmission where pathogens come from a reservoir in the environment. We then compared these two models using the Akaike Information Criterion (AIC), to investigate which model fits the data best. We carried out these steps for both herds.

Additionally, we estimated the corresponding recovery rate, *α*, using the following equation [as described in Dalen et al. (8)]:

(2) $$log\hat{(}C_{m})=\log\left(\alpha \right)+\log\left(I_{int}\right)$$

where log(Cm)^ accounts for the expected log number of recovered quarters per monthly sampling interval, *α* is the estimated recovery rate, and *I*_*int*_ is the number of infectious quarter days at risk per monthly interval. We calculated Wald 95% confidence limits of the transmission and recovery rate using the *confint* function in R.

Kirkeby et al. (20) proposed two methods for estimating the transmission rate. Both ways assume that the system is in equilibrium. These two methods do not require calculating the number of quarter days at risk for each sampling interval, but only the numbers of animals present in each state (*I*_*N*_, *I, S*, and *N*) at each sampling time are required to estimate the transmission rate. The new methods yield an estimate of the transmission rate per sampling interval compared to the Poisson regression, which provides one combined transmission rate (20). However, to inspect the robustness of the Poisson regression, we performed this analysis for each sampling interval, obtaining one estimate of the transmission and recovery rates per interval.

For method 1, we estimated the transmission rate per sampling interval using the following equation:

(3) $$\begin{array}{c} \beta =\;\frac{-log(1-\frac{I_{N}}{I})}{\frac{T\cdot S}{N}} \end{array}$$

Where *β* is the transmission parameter, *I*_*N*_ is the number of new infections per sampling interval, I is the number of infected quarters, T is the length of the sampling interval, S is the number of susceptible quarters and N is the total number of quarters after the sampling interval T. The corresponding recovery rate (α) was calculated in a similar way:

(4) $$\alpha =\frac{\log(1-\frac{I_{N}}{I})}{T}$$

with the same parameters as described in (3). Method 2 is given by the following equation:

(5) $$\beta =-\frac{1}{T}\log\left[1-I_{N}\left(\frac{1}{I}+\frac{1}{S}\right)\right]$$

Where the abbreviations are the same as described for method 1. However, method 2 allows for multiple infections and spontaneous recovery between sampling intervals Kirkeby et al. (20). The corresponding recovery rate (α) is defined by:

(6) $$\alpha =\;\frac{\log(1-I_{N}\left(S^{-1}+I^{-1}\right))}{T\left(1+\frac{I}{S}\right)}$$

with the same parameters as described above. Confidence limits for the transmission and recovery rates per sampling interval were derived by bootstrapping for these two methods. We sampled from all quarters 1,000 times with replacement. When sampling quarters, all data points over time for each selected quarter where used. By this, we obtained 1,000 estimates of the transmission and recovery rates for each sampling interval. We then calculated the 95% confidence intervals from the bootstrapped estimates of the transmission and recovery rates.

### Basic Reproduction Number

We further evaluated the epidemiological fitness of *Corynebacterium* spp. in a completely susceptible population by the basic reproduction number, *R*_0_, given by [adapted from May (21)]:

(7) $$R_{0}=\beta \cdot \tau$$

where *β* is the transmission rate, and τ is the duration of infection and the inverse recovery rate (*α*). Here, we used the 1,000 estimates of the transmission and recovery rates from the bootstrapping and took the mean of these estimates per method and estimated the *R*_0_. The 95% confidence limits were also calculated from these estimates.

## Results

### Descriptive Statistics

**Figure 1:**
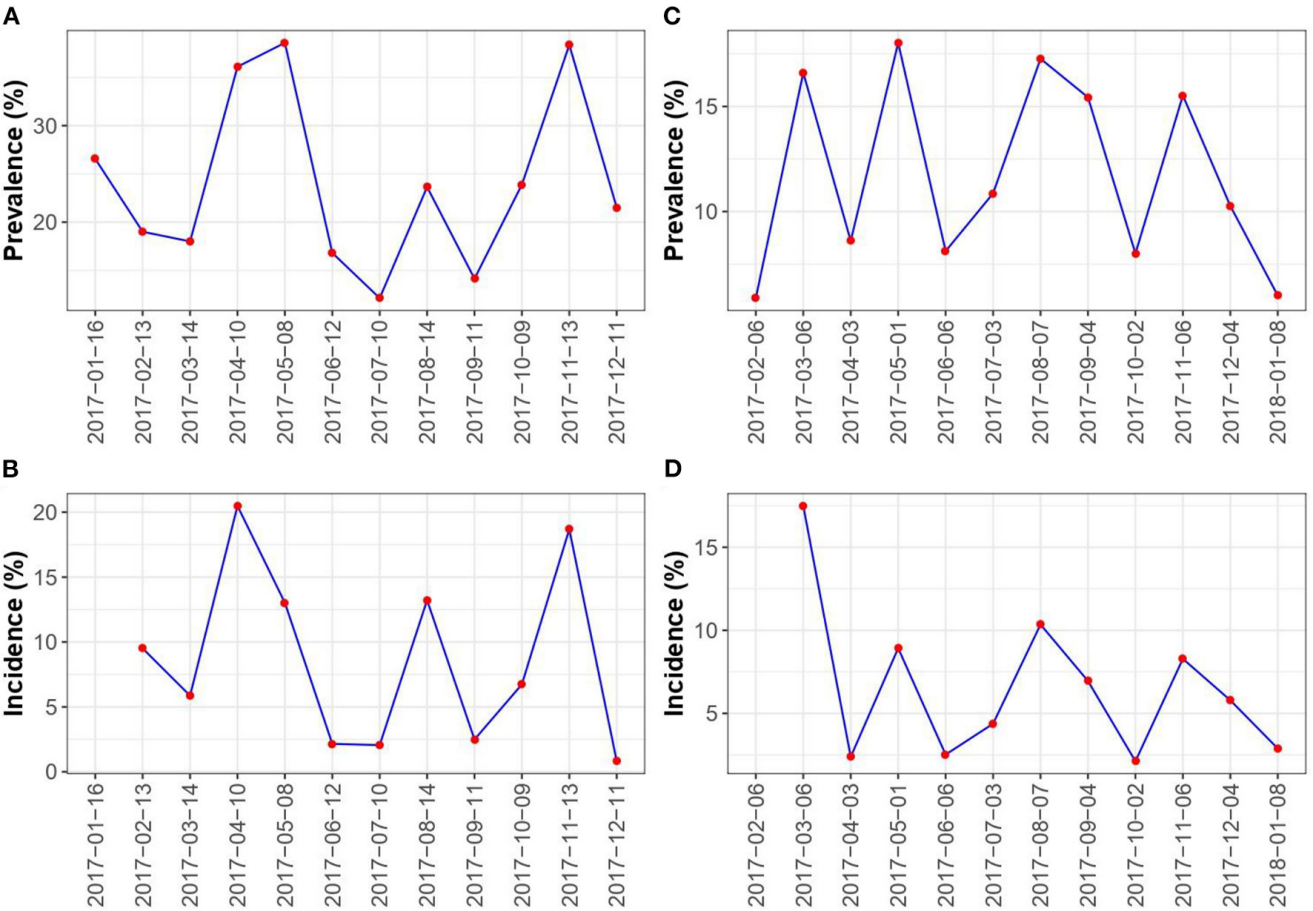
Prevalence and incidence. This figure shows the estimated prevalence and incidence of *Corynebacterium* spp. infections over time in the two dairy cattle herds in this study [**(A,B)** Herd 1, **(C,D)** Herd 2].

In herd 1, 4,665 quarter milk samples were collected from 214 lactating cows (Table 1). Detailed description can be found in Supplementary Tables 1, 2. We corrected 126 and 141 missing records to positive in herd 1 and 2, respectively, as described above. Likewise, we corrected 202 and 567 missing records to negative in herd 1 and 2, respectively. The mean quarter-level prevalence was 24% (range = 12.1–38.6) (Figure 1A). During the 12-mo study period, 320 new infections with *Corynebacterium* spp. were detected. The mean quarter-level incidence was estimated to be 8% (range = 0.8-20.5) (Figure 1B). The mean number of recovered quarters was estimated to be 34.2 per sampling interval. In herd 2, 10,381 milk samples were collected from 443 lactating cows. The mean quarter level prevalence was estimated to be 11.7% (range 5.9–18) (Figure 1C). In total, 508 new infections with *Corynebacterium* spp. were found during the study period. This corresponds to a mean quarter level incidence of 6.5% (range 2.1–17.4) (Figure 1D). The mean number of recovered (cured) quarter infections was 51.6 per sampling interval.

### Transmission and Recovery Rates

For both herds, we compared the fit of a model where transmission was dependent on the proportion of infected animals in the herd at the sampling points (Equation 1) with the model describing a fixed transmission rate, as described above. In both herds, the former model had lower AIC (215.4) than the latter (261.8), suggesting a contagious component in the transmission of *Corynebacterium* spp., e.g., via the cluster during milking or other places in the herd.

The estimated rates are presented in Table 2. In herd 1, the mean estimated transmission rate was 0.016 cases/quarter day, and the standard estimated recovery rate was 0.012 cases/quarter day. In general, there was reasonably slight variation between the estimates using the different methods. In herd 2, the mean transmission rate was estimated to 0.018 cases/quarter day, and the mean recovery rate was calculated to be 0.016 cases/quarter day (Table 2).

**Table 2:** Estimated transmission rate, recovery rate and R_0_ and 95% confidence intervals for the three methods in each of the study herds.

**Herd**	**Herd/rate**	**Poisson regression**	**Method 1**	**Method 2**	**Mean**
1	Transmission rate	0.016 (0.0104–0.0240)	0.015 (0.0018–0.0362)	0.018 (0.0018–0.0495)	0.016
	Recovery rate	0.014 (0.0099–0.0198)	0.011 (0.0014–0.0239)	0.012 (0.0015–0.0324)	0.012
	R_0_	1.13 (1.02–1.23)	1.34 (1.30–1.38)	1.34 (1.30–1.38)	1.27
2	Transmission rate	0.018 (0.0124–0.0262)	0.019 (0.0088–0.0324)	0.020 (0.0089–0.0361)	0.018
	Recovery rate	0.017 (0.0125–0.0247)	0.016 (0.0080–0.0274)	0.017 (0.0082–0.0305)	0.016
	R_0_	1.03 (0.94–1.11)	1.14 (1.13–1.16)	1.14 (1.13–1.16)	1.10

*The mean estimate of all three methods is shown in the far right column. The units for transmission rate and recovery rate is cases/quarter-day*.

**Figure 2:**
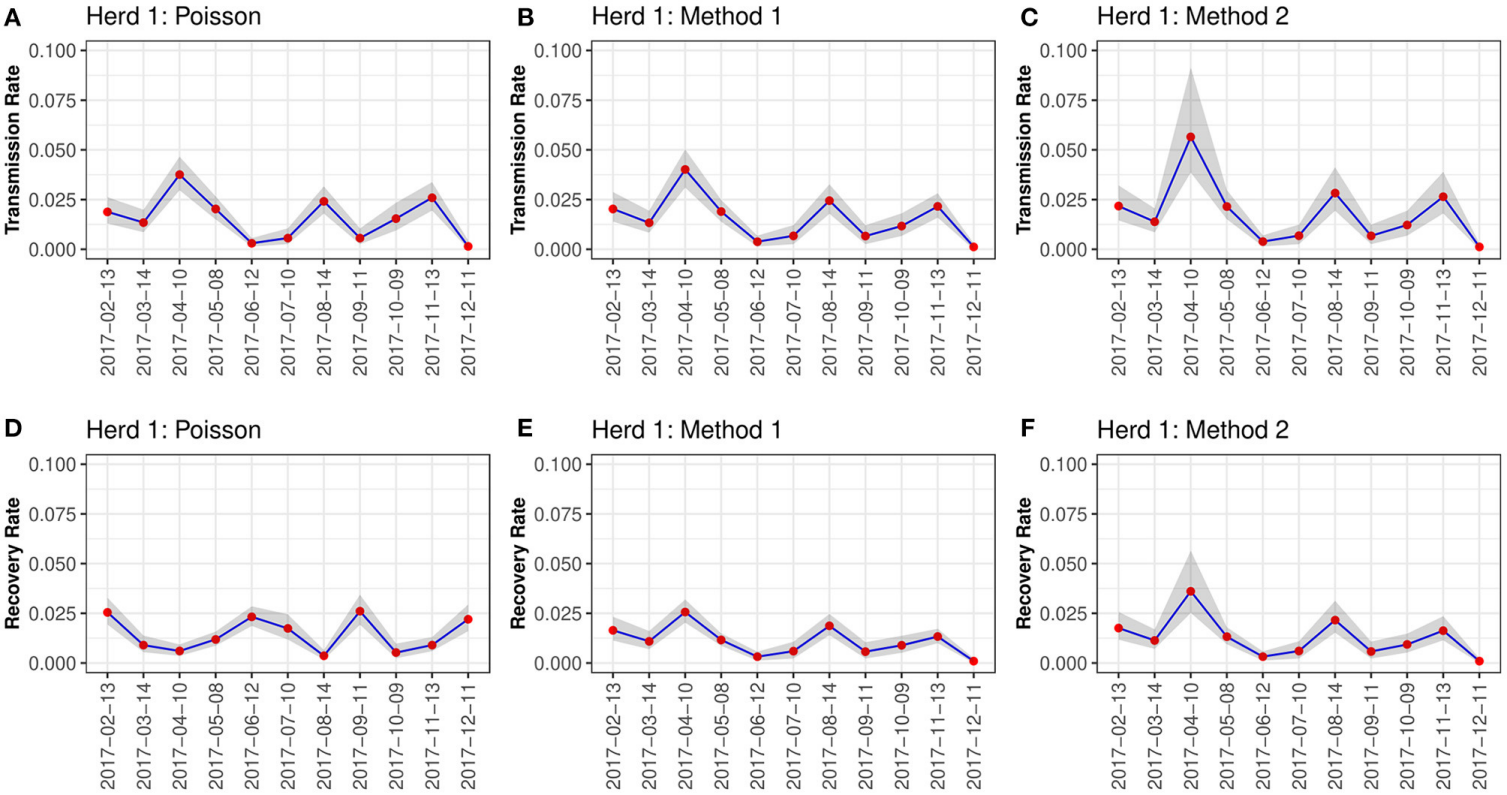
Rates for Herd 1. This figure shows the estimated transmission and recovery rates for *Corynebacterium* spp. over time during the 12-mo sampling period for herd 1 in this study. Three methods were used to calculate the estimates; Poisson regression **(A,D)**, Method 1 **(B,E)**, and Method 2 **(C,F)**.

**Figure 3:**
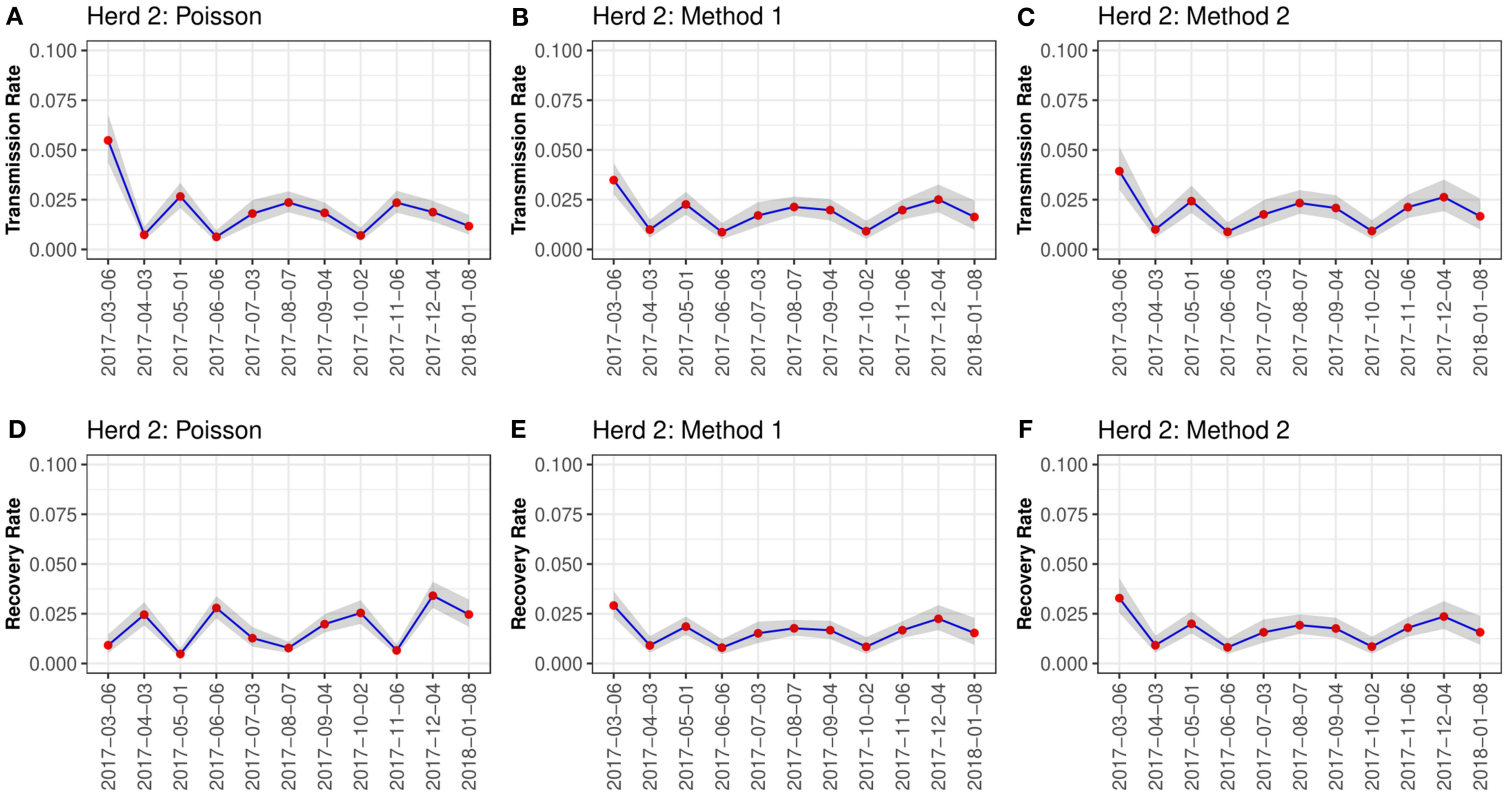
Rates for Herd 2. This figure shows the estimated transmission and recovery rates for *Corynebacterium* spp. over time during the 12-mo sampling period for herd 2 in this study. Three methods were used to calculate the estimates; Poisson regression **(A,D)**, Method 1 **(B,E)**, and Method 2 **(C,F)**.

When visually inspecting the estimated transmission and recovery rates per sampling interval for herd 1, all three methods followed similar transmission patterns (Figure 2). Generally, both transmission and recovery rates peaked in April, August, and November. However, the temporal way in recovery rate using Poisson regression appeared different from method one and method 2 with a shift in the temporal peaks (Figure 3). In herd 2, we observed that all three methods followed similar transmission and recovery patterns, except for the recovery rate estimated with Poisson regression that differed in the temporal peaks, identical to the findings for Herd 1. For all three methods, we observed that the transmission rate varied during the 12-mo sampling period, with an initial decrease, then a plateau from July to October when it decreased again before increasing again in November onwards before a final dip in January. The recovery rate followed the same course for method one and method 2 (Figures 3E,F). The estimates using Poisson regression were different from the two other methods (Figure 3).

### Basic Reproduction Number

The estimated basic reproduction number, *R*_0_, for herd 1 and 2 for the different methods are presented in Table 2. In herd 1, it was estimated to be 1.13 (95% CI: 1.02–1.23) using the Poisson regression method (Table 2). This indicates that each infectious quarter would infect 1.13 other quarters on average during its infectious period in a completely susceptible population. For method 1 and 2, the estimated *R*_0_ was 1.34 (95% CI: 1.30–1. 38) and 1.34 (95% CI: 1.30–1.38), respectively. The estimated mean *R*_0_ of the three methods was 1.27. In herd 2, the R0 was 1.03 (95% CI: 0.94–1.11) using the Poisson regression method (Table 2). The *R*_0_ for methods 1 and 2 were estimated to 1.14 (95% CI: 1.130–1.16), and the mean *R*_0_ of the three methods was 1.10 (Table 2).

## Discussion

The transmission and recovery parameters are vital measurements in decision support tools that are used to predict the cost-effectiveness of strategies to control and prevent IMI-causing pathogens. We investigated the transmission dynamics of *Corynebacterium* spp., in two Danish dairy herds, comparing models for direct (contagious) transmission with models for indirect (environmental) transmission. The contagious models performed better for both herds, indicating a contagious component involved in transmission. This supports Dalen et al. (8) findings who found similar transmission patterns for *Corynebacterium* spp. in two U.S. dairy herds.

The prevalence of *Corynebacterium* spp. in dairy herds seems to vary considerably across the world. A Swiss study reported a quarter-level prevalence for *Corynebacterium bovis* at 14% (95% C.I. 12.9–15.0%) based on 4,227 quarters samples from 99 herds (22). Other studies have reported varying prevalence of *Corynebacterium* spp. including Argentina (5.2%) (23), Jordan (5.3%) (24), Canada (19.9%) (25), and U.S. (9.1–23.9%) (8). We found a mean quarter-level prevalence at 24 and 8% (Figure 1), and thus our estimates are very similar to that of Dalen et al. (8).

In our study, the estimated mean transmission rates of *Corynebacterium* spp. in herd 1 and 2 were 0.016 and 0.018 cases per quarter day, respectively, highlighting the similarity of the two herds (Table 2). As previously described, the Poisson regression model is the most common method for estimating transmission and recovery rates in studies estimating the transmission rate of IMI causing pathogens. We estimated the transmission rates with Poisson regression for herd 1 and 2 at 0.016 and 0.018 cases per quarter day, respectively (Table 2). This difference, although not significantly different, could arise from the higher number of cows per milking parlor slot in Herd 2 than in Herd 1. Similarly, also using Poisson regression, Dalen et al. (8) estimated the transmission rates of *Corynebacterium* spp. in two comparable U.S. dairy herds to 0.018 and 0.023 cases per quarter-day. In addition, Dalen et al. (8) found that the recovery rates for farm A and B were 0.0122 and 0.0202, respectively. Our study estimated the recovery rate for herd 1 and 2 to 0.012 and 0.016, corresponding to a mean duration of infection of 83 and 63 days, for Herd 1 and 2, respectively (Table 2). Therefore, our results are in congruence with both the transmission and recovery rates found by Dalen et al. (8). Zadoks et al. (26) studied *Staphylococcus aureus* in three different herds and found recovery rates for untreated subclinical infections at 0.0052, 0.0157, and 0.0119 cases/quarter-day, which is similar to the findings for *Corynebacterium* spp. by Dalen et al. (8) and the present study.

Our estimated rates are similar across the three methods, indicating that these methods can be used interchangeably. However, the estimated rates and their confidence intervals varied between the methods used in this study. This highlights that estimation methods are sensitive to noise in the data, and that more than one method should preferably be used to increase the certainty of the estimated rates. However, the results are still only estimates of the truth. The estimated rates also vary over time during the study period (Figures 2, 3). Some of this variation comes from the cows' continuous dynamic in the herd, where new cows are dried off and calving for each sampling point. Generally, the prevalence seemed to increase in herd 1 in spring and autumn, whereas this pattern was not clear in herd 2. The prevalence was higher in herd 1, whereas the variation in prevalence between sampling points was lower in herd 2. The higher prevalence in herd 1 in spring and autumn are likely be driven by simultaneous spikes in incidence at these time points. This result is in contrast to the findings of Koivula et al. (27) who described the general seasonal trend in prevalence of *Corynebacterium* spp. in 4635 Finnish dairy herds. They found a minor peak in prevalence in February and March, and then elevated prevalence from June to October. In the present study, the prevalence and incidence generally displayed the same trends over time within each herd (Figure 1). Furthermore, these varied over time along with the transmission rates estimated for each herd (Figures 2, 3). An interesting result is that the estimated recovery rate seems to follow the transmission rate over time, except for the recovery rates estimated with Poisson regression. The temporal dynamics should be explored further in future studies to fully understand the differences between the three methods regarding the recovery rate. Method 1 and method 2 estimate the recovery rates based on an assumption of equilibrium in the system, which might be an uncertain assumption in a herd with continuous replacement. All three methods are challenged by the lack of information on the animals that are not sampled at each time point. Therefore, handling this in different ways will result in other estimates.

The transmission dynamics of *Corynebacterium* spp. were assessed for each method by estimating the R_0_. Using Poisson regression, we obtained an *R*_0_ of 1.13 and 1.03 for herd 1 and 2, respectively (Table 2). Similarly, Dalen et al. (8) estimated an R_0_ of 1.18 and 0.98 for two herds. The mean estimates of R_0_ from Method 1 and 2 were higher than for the Poisson regression. Although not significant, this is caused by the higher recovery rate estimated using Poisson regression. In addition, we observed temporal differences in the transmission of the pathogens between the two herds, in which there were spikes in the transmission in herd 1 in April and August and again in November, while the transmission was almost uniform from February in herd 2 (Figures 2, 3).

It is important to note here that we were only able to consider *Corynebacterium* spp. as a group and that the species composition will impact the transmission dynamics for *Corynebacterium* spp. Therefore, some of the pathogens in this group may be contagious, and others are environmental. The only remedy to explore this further would be identifying all pathogens down to species or strain level and then conducting the estimations. Ideally, this should be addressed in future studies. Another aspect of this is the economic value of reducing the prevalence of *Corynebacterium* spp. Gröhn et al. (5) found an association between *Corynebacterium* spp. and reduced milk production for infected dairy cows. However, the reduction in milk was less than for other pathogens and should be weighed by the costs of implementing control actions to improve udder health. These control actions can be more or less effective depending on the herd and must be considered along with the overall udder health management. For instance, post-milking teat dipping was not used in herd 2 in this study, but the estimated transmission rate was not significantly higher. However, as there are many other differences between the two herds, evaluation of single control actions is not possible in this study. This also highlights that udder health management should be herd specific. Control measures that may work well in one herd, may not in another herd, and hence these measures should be continually assessed.

## Conclusion

This study explored the transmission dynamics of *Corynebacterium* spp. IMI at quarter-level in two Danish dairy cattle herds. We found that the best model to describe the spread of *Corynebacterium* spp. between the cows was direct transmission. This suggests that farmers should consider hygiene around milking to decrease the transmission of *Corynebacterium* spp. Estimates for the transmission dynamics of *Corynebacterium* spp. in dairy herds are important to develop proper control actions. Future research should focus on investigating these dynamics in more herds, and possibly on species or strain level.

## Data Availability Statement

The original contributions presented in the study are included in the article/Supplementary Material, further inquiries can be directed to the corresponding author/s.

## Ethics Statement

Ethical review and approval was not required for the animal study because this was not needed for this type of study. Written informed consent was obtained from the owners for the participation of their animals in this study.

## Author Contributions

TH coined the idea for this study. GC carried out initial analyses and wrote the first draft. CK conducted the final analyses and wrote the final manuscript. All authors participated in design of the study and analyses and in writing the manuscript.

## Conflict of Interest

The authors declare that the research was conducted in the absence of any commercial or financial relationships that could be construed as a potential conflict of interest.

## Publisher's Note

All claims expressed in this article are solely those of the authors and do not necessarily represent those of their affiliated organizations, or those of the publisher, the editors and the reviewers. Any product that may be evaluated in this article, or claim that may be made by its manufacturer, is not guaranteed or endorsed by the publisher.
