# Supplementary material for: Transmission Dynamics of Corynebacterium spp. Within Two Danish Dairy Cattle Herds
Source: Front Vet Sci. 2021 Aug 23;8:735345. doi: 10.3389/fvets.2021.735345 (PMC8419450; doi:10.3389/fvets.2021.735345)
Supplement: Supplementary file 1 [file Data_Sheet_1.pdf]

SUPPLEMENTARY INFORMATION

Table S1. *Corynebacterium* spp. records and results from Herd 1, showing the numbers of susceptible and infected quarters, and the numbers of newly infected and recovered quarters per time interval.

Date	Susceptible quarters	Infected quarters	New quarter infections	Recovered quarters
16-01-2017	271	98	-	-
13-02-2017	358	84	31	55
14-03-2017	369	81	22	18
10-04-2017	269	152	76	17
08-05-2017	269	169	47	43
12-06-2017	376	76	8	85
10-07-2017	377	52	8	29
14-08-2017	323	100	48	9
11-09-2017	328	54	8	46
09-10-2017	274	86	19	8
13-11-2017	220	137	51	25
11-12-2017	281	77	2	42

Table S2 *Corynebacterium* spp. records and results from Herd 2, showing the numbers of susceptible and infected quarters, and the numbers of newly infected and recovered quarters per time interval.

Date	Susceptible quarters	Infected quarters	Newly Infected quarters	Recovered quarters
06-02-2017	496	31	-	-
06-03-2017	703	140	78	15
03-04-2017	850	80	18	69
01-05-2017	764	168	68	14
06-06-2017	860	76	19	96
03-07-2017	804	98	33	26
07-08-2017	800	167	77	30
04-09-2017	822	150	56	72
02-10-2017	931	81	17	70
06-11-2017	860	158	70	22
04-12-2017	935	107	50	104
08-01-2018	830	53	22	50
